# Association of *Candida sp.* with the Degrees of Dysplasia and Oral Cancer: A Study by Calcofluor White under Fluorescent Microscopy

**Published:** 2017-10-01

**Authors:** Sandhya Tamgadge, Avinash Tamgadge, Aswathy Pillai, Mayura chande, Siddharth Acharya, Narayan Kamat

**Affiliations:** *Dept. of Oral and Maxillofacial Pathology and Microbiology, D .Y. Patil Dental College, school of dentistry l Sector -7, NerulNavi Mumbai , Maharashtra , India*

**Keywords:** *Candida sp.*, Calcofluor White, Fluorescent Microscopy, Histopathology, Leukoplakia, Dysplasia, Oral Carcinoma

## Abstract

**Background and objective:**

*Candida albicans* (*C. albicans*) play a significant role in oral mucosal carcinogenesis. It can be identified using various techniques in cytological smears. But, very few studies have been conducted on histopathological sections using calcofluor white M2R under fluorescent microscopy. Additionally, detection and quantification of *Candida* colonies and its correlation with various grades of oral leukoplakia and oral carcinomas have not been explored much.

**Methods:**

The current retrospective study included 80 samples from archives consisting of 60 samples in the study group (10 cases each of mild, moderate, and severe epithelial dysplasia (totally 30) and 30 cases of oral carcinoma). Sections were stained with calcofluor white (CFW) and 10% KOH for the observation under fluorescent microscopy and correlated with different grades of oral leukoplakia and oral carcinomas. Chi-square test was used in SSPS software to study the presence and absence of *Candida* sp. in different groups.

**Results:**

The study groups of oral carcinoma and dysplasia showed a significant association with *Candida* sp. (P=0). When carcinoma was compared with each grade of dysplasia, except mild dysplasia (P=4.4E-05), both moderate (P=0.402195) and severe dysplasia (P=0.558746) showed an insignificant P-value. When the groups of mild (13.3%), moderate (30%), and severe (33.3%) dysplasia were considered independently, the incidence of *Candida* sp. increased as the grade of dysplasia increased. The number of colonies have been counted and the maximum number of colonies have been observed in carcinoma and the least have been observed in mild dysplasia.

**Conclusion:**

A significant association of *Candida* colonies with epithelial dysplasia and oral cancer was established. Further, CFW was found a promising candidate to identify *Candida* colonies in tissue sections using fluorescent microscopy.

## Introduction

 The incidence of oral precancers and carcinomas is rising at an alarming rate in India and causing major health care concerns. Sometimes, oral cancers are reported in patients with questionable habits and the role of microbes have been postulated by the researchers. *Candida* sp. have the normal oral microflora proved to be strongly associated with carcinogenesis; therefore, its detailed etiopathogenesis needs analysis. There is plethora of literature, which point to a strong association of *Candida* sp. with oral cancer, but there are scarcely any studies establishing the association of *Candida* sp. in dysplastic alteration in the epithelium (1).

Various techniques are used to detect *Candida* sp. in oral cavity (1) in which cytological study is the most common method, but there are very few studies performed on the histopathological sections to evaluate the invasive property of this fungal infection (2). Rashmi et al., did a similar study, but the degree of dysplasia was not correlated with the presence of *Candida* sp. (3). Additionally, many studies used the periodic acid–Schiff (PAS) staining method (3,4) to detect *Candida* sp. But, calcofluor white (CFW) stain under fluorescent microscopy and its correlation with different grades of dysplasia are not analyzed so far (3). The current article reported the same which makes the study unique.

## Materials and methods

The current retrospective study was conducted in D.Y. Patil School of Dentistry Nerul navi, Mumbai, India. Samples were randomly selected from the archives. The control group consisted of 20 samples with normal gingiva. The study group consisted of 60 samples (10 samples each from diagnosed cases of mild, moderate, and severe dysplasia (totally 30), as per 2005 World Health Organization (WHO) classification), and 30 samples with oral carcinoma).

 Clinical details were not considered in the study due to incomplete data available in most of the biopsy requisition forms.

 Since *Candida* sp. has the affinity for epithelium, previously H & E stained sections of each sample were viewed for adequacy of the epithelium, and selected. Sections were deparaffinized, hydrated, and flooded with 1 or 2 drops of CFW and 10% KOH for 1minute, and then, cover-slipped and examined under fluorescent microscopy. Although the solution was relatively stable, it was kept in actinic (red) glassware to prevent photoisomerization and degradation. The procedure was performed in dark room, and slides were immediately placed inside the slide box to keep them away from light. All sections were examined by the inverted fluorescence microscopy (LEICA DM 1000 LED) equipped with mercury light source and an ultraviolet (UV) filter cassette.

The number of *Candida* hyphae and colonies were counted by 2 experienced oral pathologists who were blindfolded. The entire epithelium was studied under 4X, 10X, and 40X magnification and hyphae were located and counted. If they were present in groups, they were considered as colonies. The *Candida* sp. counts were correlated with histopathological grades of oral leukoplakia and oral carcinoma. All the findings were tabulated and statistically analyzed.

## Observations and results

Fungal hyphae were predominantly found in the superficial part of epithelium. *Candida* hyphae, spores, and colonies fluoresced brightly ‘blue-white’ in a blue background when exposed to UV light. 

**Figure 1:**
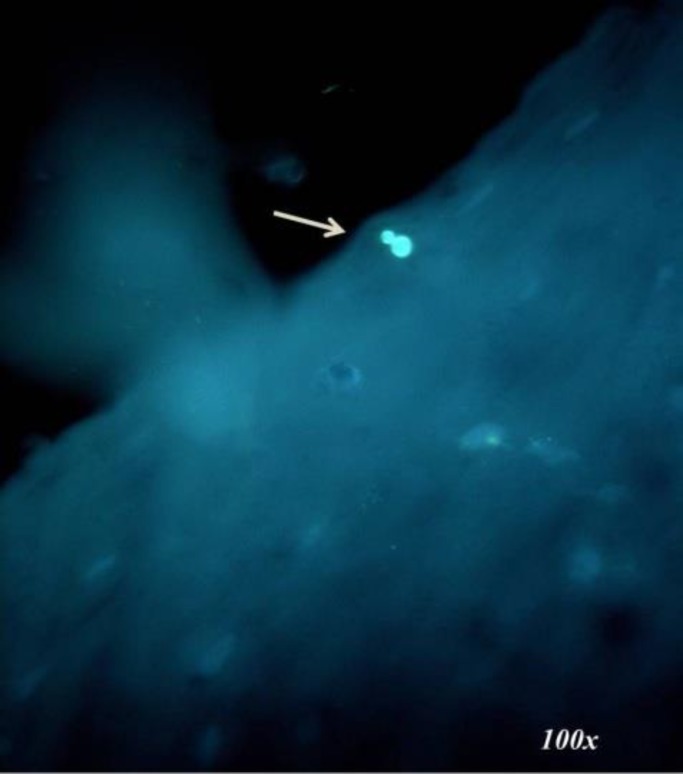
Photomicrograph of a Candida spore in the control group

Among 20 samples in the control group, 2 samples showed the presence of *Candida* sp. (Figure 1).

**Figure 2:**
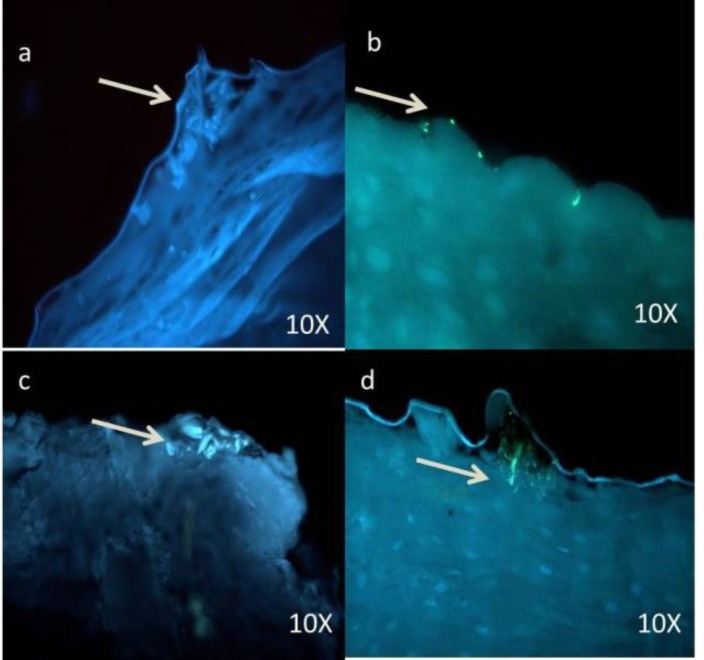
Photomicrograph shows the sparse distribution of Candida sp. in superficial part of epithelium in the mild dysplasia group

**Figure 3:**
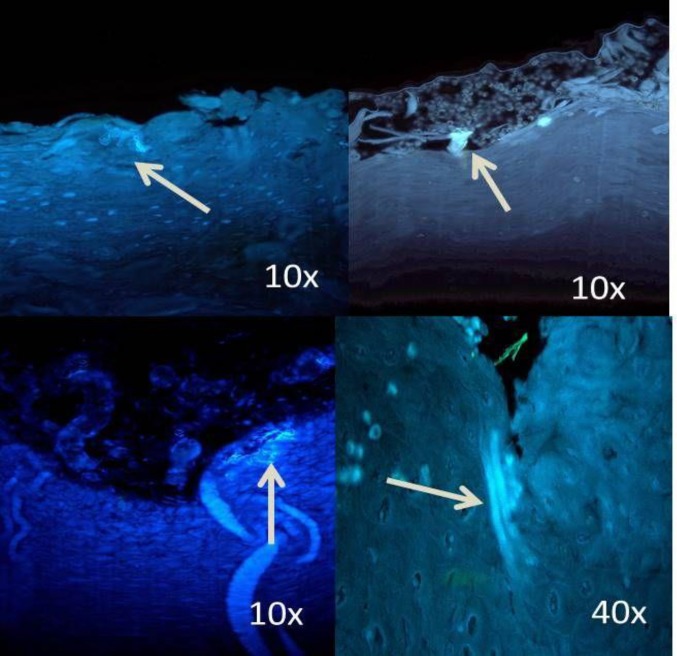
Photomicrograph of comparatively more number of Candida sp. in moderate dysplasia, compared with mild dysplasia.

**Figure 4:**
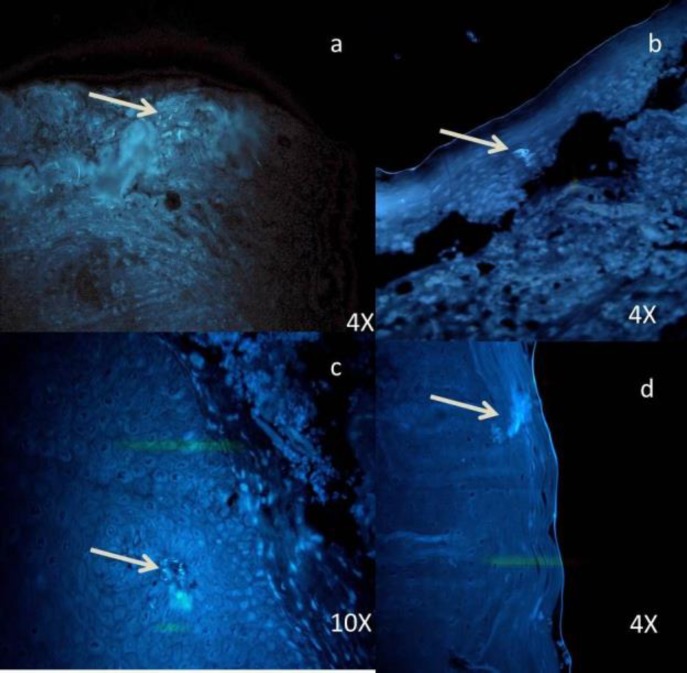
Photomicrograph of abundant Candida sp. even in the deeper layers of epithelium in severe dysplasia

 Among different groups of dysplasia, colonies were mostly distributed in moderate and severe when compared to mild dysplasia (Figures 2, 3, 4). 

**Figure 5:**
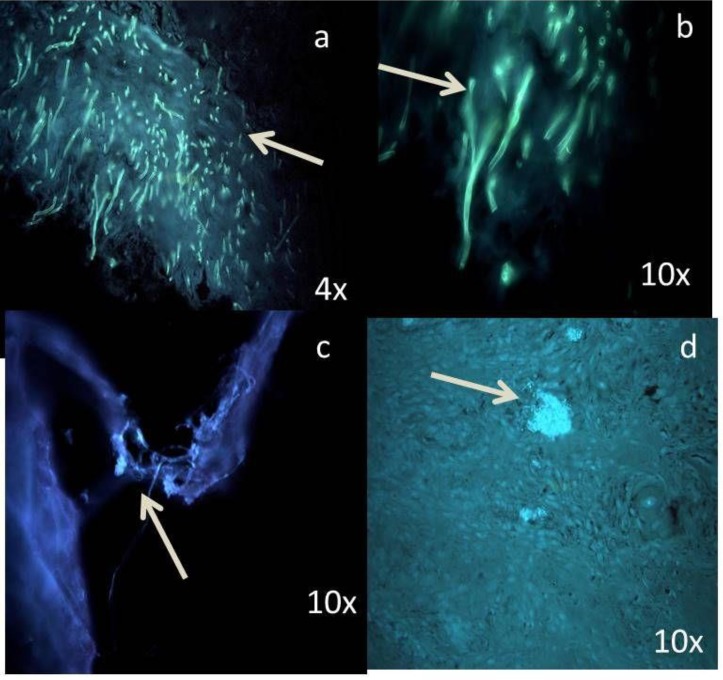
Photomicrograph shows dense multiple foci of Candida sp. (a, b ,c) in verrucous carcinoma; (d) Candida sp. even in invading islands

In the oral carcinoma group of 30 samples, 4 samples were verrucous carcinoma (Figure 5), which showed dense and evenly distributed fungal hyphae. 

**Figure 6:**
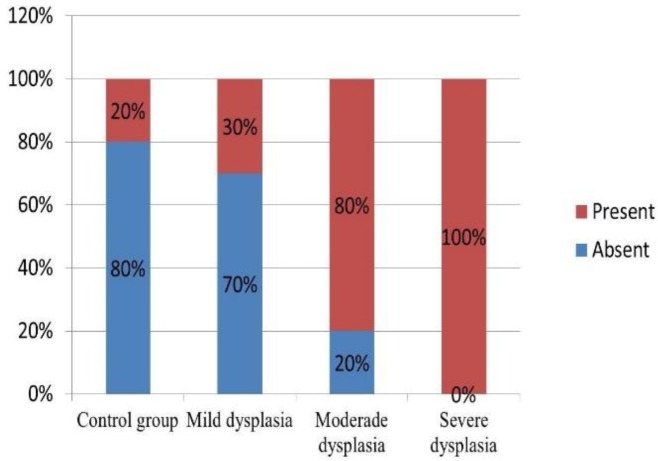
Comparison of the Presence of Candida sp. between the Control and Dysplasia Groups

Presence of *Candida* sp. was observed in 30% of mild dysplasia and in 80% of moderate, and all samples (100%) of severe dysplasia (Figure 6).

**Figure 7:**
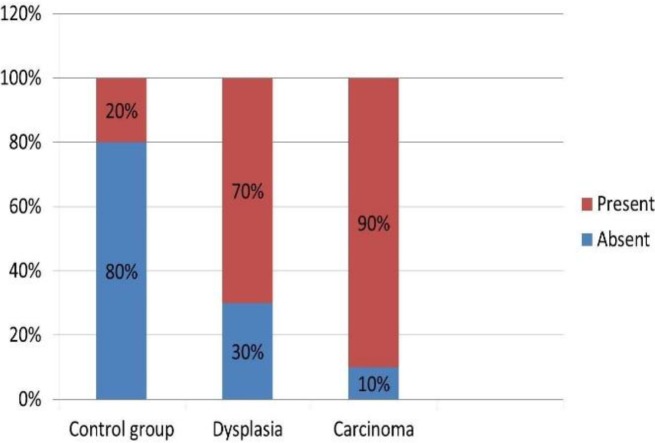
Comparison of the Presence of Candida sp. between the Control and Study Groups

Also, when dysplasia group was considered as a whole, 70% of the samples showed the presence of *Candida* sp. In the carcinoma group, 90% of the samples showed the presence of *Candida* sp. (Figure 7).

 All data were tabulated and statistically analyzed by Chi-square test in SPSS software.

 In comparison with the control group, the study group of cancer and dysplasia showed a significant association with the presence of *Candida* sp. (P=0) (Table 1).

**Table 1:** The Presence of *Candida* sp. between the Study and Control Groups

Yes	No		Marginal Row Totals
52 (86.67%)	8 (13.33%)	**Study**	60
2 (10%)	18 (90%)	**Control**	20
54	26	**Marginal column totals**	80 (grand total)

Chi-square = 40.1899; P-value = 0.0

The results were significant at P<0.05.

The above table showed the distribution of samples with cells present in 2 groups. The Chi-square test was used to analyze the association between the presence and absence of cells in the control and study (dysplasia and carcinoma) groups. The results indicated a significant difference in the number of cells in the study and control groups (Chi-sqsqare = 40.1899, P = 0.0).

When a comparison of control group with several grades of dysplasia was conducted except for mild dysplasia (P=0.052808***),*** both moderate (p=2E-6) and severe dysplasia (p= *2E-06*) showed a significant P-value (Table 2).

**Table 2:** The Presence of *Candida* sp. Between the Control and Dysplasia groups

	Yes	No	Marginal Row Totals
Dysplasia	23 (76.67%)	7 (23.33%)	30
Control	2 (10%)	18 (90%)	20
Marginal column totals	25	25	50 (grand total)

Chi-square = 21.3333; P-value = 0.000004

The results were significant at P<0.05

Table 2 shows the distribution of cells in the 2 groups. The Chi-square test was used to analyze the association between the presence and absence of cells in the control and dysplasia groups. The result indicated a significant difference in the cells between the control and dysplasia groups (Chi-square = 21.3333, P=0.000004).

 When carcinoma was compared with each grade of dysplasia, except mild dysplasia (P=4.4E-05), both moderate (P=0.402195) and severe dysplasia (P=0.558746) showed insignificant P-values (Table 3).

**Table 3:** The Presence of *Candida* sp. between the Dysplasia and Carcinoma Groups

	Yes	No	Marginal Row Totals
Dysplasia	23 (76.67%)	7 (23.33%)	30
Carcinoma	29 (96.67%)	1 (3.33%)	30
Marginal column totals	52	8	60 (grand total)

Chi-square stastistic = 5.1923; P-value = 0.022687

The results were significant at P<0.05.

Table (3) shows the distribution of cells in the 2 groups. The Chi-square test was used to analyze the association between the presence and absence of cells in the dysplasia and carcinoma groups. The results indicated a significant difference in the cells present between the dysplasia and carcinoma groups (Chi-square = 5.1923, P = 0.022687).

**Figure 8:**
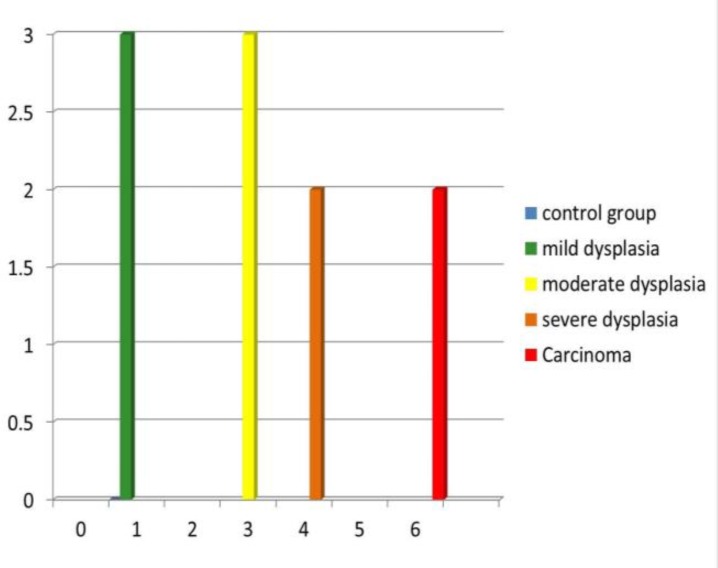
Comparison of the Number of Colonies between the Control and Study Groups

The maximum number of colonies was 6 in the carcinoma group and the minimum number of colonies were 1, in the control and mild dysplasia groups (Figure 8). 

## Discussion

Oral cancer is a multifactorial disease and its etiopathogenesis is incompletely understood (5). Therefore, the literature focused on the role of *Candida* sp. as the potential fungi responsible for oral carcinogenesis. There are ample reports, which state that among the potentially malignant lesions, oral leukoplakia has the highest rate of incidence and malignant transformation. Additionally, leukoplakia along with *Candida* infections aids to its severity (5, 6) .Therefore, only leukoplakia was included amongst the potentially malignant group in this study.

*Candida* *albicans* are the dwellers of normal flora of oral and gastrointestinal tract (7), the key species associated with human oral mycoses, and the most lethal among pathogenic *Candida* species (8). Cytological studies are believed to be the best method to identify *Candida* species (9). But, very few studies have been conducted on the histopathological sections using florescent microscopy with CFW staining and correlated with different grades of oral leukoplakia and oral carcinomas, which makes this study unique.

The ability of *Candida* species to transform a blastospore to a hyphae phase and later into a germ tubes, which indicate the onset of hyphal growth in *Candida*, is the possible factor in the pathogenesis of candidiasis (10). In the current study, similar forms of blastospores and hyphae were observed.

 Various special stains such as PAS and Gomori methenamine silver (GMS) are used to detect *Candida* sp. in histopathological examinations (11), but CFW is a quick and easy way to identify fungal hyphae^. ^(3). The use of CFW in clinical mycology was first described by Hageage and Harrington (12). It is a fluorochrome that binds with cellulose and chitin contained in the cell walls of fungi and other organisms and exhibits fluorescence when exposed to long-wavelength UV and short-wavelength visible light(13,14). CFW, a disodium salt of 4,4'-bis-[4anilino-bis-diethyl-amino-S-tri-azin-2-ylamino]-2,2'-stilbene-disulfonic acid, is a colorless dye that is used in the textile and paper industries as a whitening agent (12,14). When viewed under a fluorescent microscopy, fungal elements stained with CFW are sharply delineated from surrounding tissue and easily identified (15). Fluorescents can be either apple-green or blue-white color when exposed to UV light and it depends on the filter system used (13, 16,17). It can be used on fresh smears, frozen, fixed, paraffin embedded, and clinical specimens (14). CFW staining should be performed in a dark room to prevent photoisomerization and degradation (3). Therefore, precaution was taken while staining and carrying the slides to the viewing area in a covered slide box (3). But, Rashmi et al., in their study stated that CFW remains relatively stable and should be kept in actinic (red) glassware. Along with CFW, 1 or 2 drops of KOH was also used as a clearing agent, which aids to dissolve the tissue cells (18).

According to Samaranayake et al., blastospore forms of *Candida* sp., round or ovoid, are usually found on the surface of epithelium, and the hyphal forms are vertically penetrating into the epithelium (19). Similar findings were observed in the current study (Figure 5). Even few samples in the control group showed the presence of *Candida* species, as it is a commensal oral microflora.

According to the literature, the density of *Candida* sp. increases with the increase in keratin deposition (20). The current study also observed similar findings as the density of *Candida* sp. was more in verrucous carcinomas. Therefore, normal gingiva was included in the control group samples, as it was keratinized.

Siddharth Kumar et al., reported no correlation between the severity of epithelial dysplasia and the presence of *Candida* species (21). In contrary, the current study showed a significant correlation between these 2 parameters, which was consistent with the results of McCullough et al. (22). Also, it was found that the presence of *Candida* sp. was directly proportional with the grade of dysplasia (23); similar significant correlation was also found in the current study. 

However, the parameters taken by Siddharth et al., consisted of potentially malignant disorders; whereas in the current study, among potentially malignant disorders, only oral leukoplakia was considered. And the positive correlation may be due to the high malignant transformation rate of leukoplakia (24).

In the current study, among the entire groups, carcinoma showed a statistically significant value for the presence of *Candida* sp., which was already reported by many researchers and was first reported in 1960’s (Cawson 1969, Williamson 1969) (25, 26).

When the samples were observed under fluorescent microscopy, various tissue components other than *Candida* species, such as cotton fibers and dust particles, were brightly fluoresced (27). Therefore, care should be taken to avoid contamination during tissue processing and staining to achieve better results. Sometime red blood cells (RBCs) can be mistaken with the spore form of the hyphae, which can be differentiated on morphological basis.

When the number of *Candida* colonies was counted, they were maximum in carcinoma and minimum in mild dysplasia. The carcinoma group, especially verrucous carcinoma, showed dense and evenly distributed colonies. This could be due to excessive keratin layers, which favor the growth of *Candida* sp. (19).

The increased number of *Candida* colonies could be also attributed to the immunocompromised state of the patient (28, 29, 30). Such fungi produce various metabolic products such as acetaldehyde nitrosamines, specific proteinases, and induction of proinflammatory cytokines, which are harmful to the host cells and thus promote carcinogenesis (31, 32).

Although various studies reported the presence of *Candida* sp. in the superficial portion/keratin layer of the epithelium, but few authors found *Candida* sp. in connective tissue (33), which could be due to the aggressive nature of the lesion where the neoplastic cells metastasize through angiogenesis and *Candida* hyphae have high affinity toward endothelial cells (34). 

Pragati et al., in their study included verrucous carcinoma in malignant group to detect its association with *C. albicans, *which was positive. Therefore, the current study also included verrucous carcinoma in oral carcinoma group and it was strongly positive (35).

 Various stains such as PAS can detect *Candida* sp. in histopathological sections, but the calcofluor is easy to use, quick in response, and can be restained. Additionally, it easily identifies all forms of *Candida* sp. in histopathological sections due to high contrast (3).

As *Candida* species are mostly observed in immunocompromised patients, a clinical correlation with more sample size should also be considered, as erythematous areas are more dysplastic. 

## Conclusion

The overall analysis of the current study revealed the significant role of *Candida* sp. in all grades of dysplasia, except for mild dysplasia, and the number of *Candida* colonies was directly proportional to the grades of dysplasia. To evaluate the presence of *Candida* species, CFW staining is superior because it is an easy-to-use and rapid method of staining and viewing both cytological smears and histopathological samples. Verrucous carcinoma has strong association with human papillomavirus (HPV), but the current study proved its association with *Candida* species too, with prominent candidal hyphae in the keratin layer. Such a study should also include the clinical parameters and its correlation with histopathological parameters to prove the pathogenicity of *Candida* lesions.

## Conflict of Interest

Author declared no conflict of interest. 

## Funding

There was no financial support to the study.

## Ethical approval

 No human or animals participated in the study. Therefore, ethical approval was not considered.
